# Circular RNA FUNDC1 improves prediction of stroke associated infection in acute ischemic stroke patients with high risk

**DOI:** 10.1042/BSR20200902

**Published:** 2020-06-18

**Authors:** Lei Zuo, Cai Li, Juan Zu, Honghong Yao, Fuling Yan

**Affiliations:** 1 Department of Neurology, Affiliated ZhongDa Hospital, School of Medicine, Southeast University, Nanjing 210009, China; 2 Department of Neurology, Rizhao Hospital of Traditional Chinese Medicine, Rizhao, 276800, Shandong, China; 3 Department of Pharmacology, School of Medicine, Southeast University, Nanjing 210009, China

**Keywords:** acute ischemic stroke, biomarker, circular RNA, risk models, stroke associated infection

## Abstract

Identifying those patients who were at high risk of stroke associated infection (SAI) for preventive antibiotic therapy was imperative for patients’ benefits, thus improving prediction of SAI was critical for all acute ischemic stroke (AIS) patients. Circular RNA FUNDC1 (circFUNDC1) has been reported to be the diagnosis and prognosis biomarker of AIS. Therefore, the present study aimed to figure out whether circFUNDC1 could be the potential predictor of SAI that could help to guide preventive treatment. In total, 68 patients were included in the study, 26 of which had infection and 42 without. Copy number of circFUNDC1 in plasma were quantified by quantitative real-time polymerase chain reaction (qPCR). Platelet spike-in experiment and correlation analysis were conducted to explore possible origins of circFUNDC1 in plasma. A significantly elevated level of circFUNDC1 was found in SAI patients compared with not infected AIS patients (*P*=0.0258). Receiver operating characteristic (ROC) curves demonstrated the prediction significance of circFUNDC1, with the area under the curve (AUC) at 0.6612 and sensitivity, specificity at 69.23%, 61.90% respectively in predicting SAI. Then, when adding circFUNDC1 in the risk model, the AUC increased from 0.7971 in model A to 0.8038 in model B. Additionally, positive correlation was observed between circFUNDC1 level and neutrophils counts. WBC and neutrophil ratios were significantly elevated in SAI patients compared with non-SAI patients. Therefore, circFUNDC1 could be used to construct a risk model for the prediction of SAI that is beneficial for AIS patients’ preventive treatment.

## Introduction

Despite advances in diagnosing, clinical management and critical care for patients with acute ischemic stroke (AIS), complications accompanied by stroke have always been a huge threat to patients’ survival and life quality. Infection is one of the major complications causing poor outcomes and death. Up to 30% of AIS patients can develop an infection within 3 days after symptom onset, while pneumonia is the most common and fatal kind among them [1]. In recent years, multiple researches have tried to figure out risk factors and preventive treatments for stroke associated infection (SAI). SAI was reported to be associated with stroke severity [2], infarct lesion [3], hyperglycemia [4], dysphagia and stroke-induced immunodepression syndrome [5].

Preventive strategies, especially such as the preventive antibiotics therapies, have been applied to SAI investigation. Though preventive antibiotic therapy reduced the risk of infection, but did not affect the distribution of functional outcome scores and did not result in an increased occurrence of adverse events [6–8], partially owing to side effects of antibiotics. Therefore, selecting those patients at high risk by significant biomarkers for preventive antibiotic therapy was imperative for patients’ benefits. Consequently, identifying possible biomarkers to predict and get early identification of SAI may negatively influence the outcome and help to reduce the health and economic burden of AIS.

Recent clinical studies have tested the efficacy of cytokines as biomarkers of SAI. Among them, higher levels of interlukin-6 and interlukin-10 are associated with the occurrence of SAI [9]. Furthermore, non-coding RNAs also have been studied, up-regulating miRNA-21 in plasma was verified to be a new biological predictor for SAI [10].

Circular RNAs have been verified to participate in multiple processes after AIS and inflammation [11]. Moreover, our previous research has demonstrated that circFUNDC1, circPDS5B and circCDC14A could be diagnosis and prognosis biomarkers of AIS [12]. Therefore, the present study aimed to figure out whether these circRNAs could be potential predictors of SAI that could help to guide preventive treatment.

## Methods and materials

### Study population

The current study was approved by the ethics committee of the Zhongda Hospital (approval ID: 2019ZDSYLL080-P01), and the participants or their legally authorized representatives provided written informed consent to participate in the study. AIS Patients who were admitted into Neurology department of Zhongda hospital within 72 h of symptom onset were included. NIHSS score were assessed by advanced neurologist on hospital arrival while patients with NIHSS score below 5 and above 20 were excluded. Furthermore, patients with active malignant diseases or other neurological and psychiatric diseases, those who underwent surgery within the last 3 months, and those who took prior medication with low-molecular or unfractionated heparin within the last month were excluded from our research too. SAI were defined as AIS patients diagnosed with infection during the first week after stroke [3]. Infections were diagnosed by the clinician according to modified Centers for Disease Control and Prevention criteria [6]. All patients involved were followed up for 3 months by telephone or re-examination.

### Patient blood sampling and processing

Blood samples from AIS patients were collected at arrival of hospital. Whole blood was drawn into EDTA-containing tubes (BD Biosciences) prior to the administration of any therapies. The samples were processed by centrifugation at 1000 × ***g*** for 10 min at 4°C. Total RNA was extracted from the plasma of every AIS patient using a miRNeasy Mini kit (Qiagen) according to the manufacturer’s protocol and was quantified using a NanoDrop ND-1000 spectrophotometer (Thermo Fisher Scientific, Waltham, MA, U.S.A.).

### Reverse transcription and qPCR assay

The total RNA of plasma was reverse transcribed with the HiScript Q RT SuperMix for qPCR Kit (Vazyme, R123-01) according to the manufacturer’s instructions. Quantitative PCR was performed on the Applied Biosystems QuantStudio 6 (Applied Biosystems) using the manufacturer’s recommended cycling conditions with SYBR Green Real-time PCR Master Mix (Vazyme, R131-01). All samples were run in duplicate. Replicates of individual samples with *C*q values > 35 were removed from the analysis. Copy numbers of circRNAs were calculated using recombinant circFUNDC1 synthesized by Genewiz. The primers of circFUNDC1 (hsa_circ_0007290, forward: CCATCTGAAGCTTGGCAAACT, reverse: TTCAACTCTCTTCCAGTCAATCT), circPDS5B (hsa_circ_0004494, forward: ATTGCTCTCCTTGCACCTGA, reverse: TCGCATGGATACAATGAATGGC) and circCDC14A(hsa_circ_0000097, forward: CCATTCTCGACTGTTTGCAGG, reverse: GACAGGAGTGCTCTGTAGGC) were synthesized by Invitrogen.

### Platelet spike-in experiment

The experiment was conducted following a protocol that had been published [13]. Briefly, whole blood from eight healthy volunteers was drawn into EDTA-plasma containers (BD). After staying at room temperature for 30 min, blood samples were centrifuged at 200 ×*** g*** for 30 min at room temperature to eliminate red blood cells. Next, the supernatant was centrifuged at 300 × ***g*** for 10 min to deplete white blood cells. Then 200 μl of supernatant was saved as platelet-rich plasma (PRP) and were kept for RNA extraction. The remainder was centrifuged at 1200 × ***g*** for 10 min to gather platelets. The remaining supernatant was platelet-poor plasma (PPP). To further purify platelets, the platelet pellet was washed twice with modified Tyrode’s buffer (134 mM NaCl, 2.9 mM KCl, 0.34 mM Na_2_HPO_4_, 12 mM NaHCO_3_, 20 mM HEPES, 1 mM MgCl_2_, pH 7.4; glucose (45 mg/50 ml) was added just before use, and the solution was warmed to 37°C in a water bath). Prostaglandin E1 and indomethacin were added during each wash at the concentrations mentioned above. The final platelet solution was again pelleted and resuspended in 1/20 of the initial PRP volume to obtain a 20× stock platelet solution. Eventually, this stock solution was used to reconstitute PPP with platelet concentrations of 5%, 50%, 100% and 200%. Subsequently, RNA was extracted using miRNeasy Mini kit (Qiagen).

### Statistical analysis

Statistical analyses were conducted with Statistical Packages for Social Sciences (SPSS), version 22. Data are expressed as the number (percentage) for categorical variables and as the mean (standard error) or median (interquartile range) for continuous variables depending on the data distribution (assessed by the Kolmogorov–Smirnoff test). For each comparison, univariate analysis was performed using the χ2 test for categorical variables and, depending on the data distribution, Student’s *t* test or the Mann–Whitney *U* test for continuous variables. Significant variables were used to construct risk models to predict SAI by logistic regression analysis. Correlations were performed by Pearson correlation analysis. Statistical significance was defined as *P*<0.05.

## Results

### Elevated level of circFUNDC1 in SAI patients and role of circFUNDC1 as a biomarker of SAI

**Figure 1:**
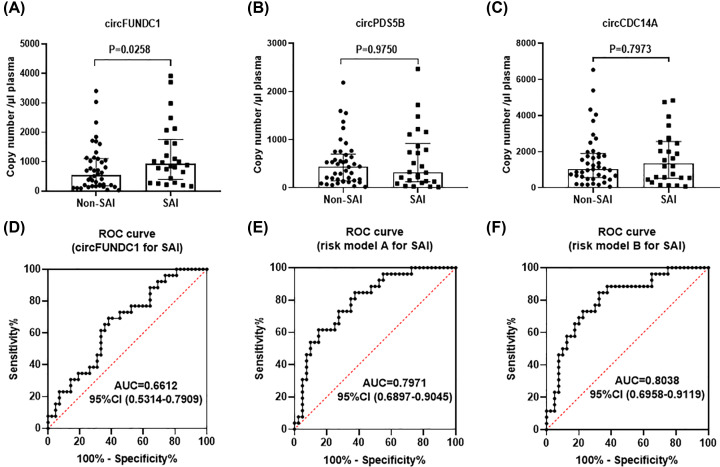
Expression of circFUNDC1, cricPDS5B and circCDC14A in acute ischemic stroke (AIS) patients and predictive value of circFUNDC1 for SAI (**A–C**) circFUNDC1, circPDS5B and circCDC14A levels in SAI patients and non-SAI patients, *n* = 26 and 42 respectively, median ± interquartile range, Mann–Whitney test. (**D**) Receiver operating characteristics (ROC) curve was calculated merely depending on circFUNDC1 level in predicting SAI. (**E** and **F**) ROC curves were calculated by risk model A and risk model B.

In total, 68 patients were included in the study, 26 of which had infection (including respiratory infection and urinary infection) and 42 without. Patient demographics and baseline characteristics are summarized in Table 1. Among them, age, NIHSS score, white blood cell (WBC) and triglycerides level on admission were significant different between these two groups. Besides, significantly elevated level of circFUNDC1 was found in SAI patients compared with not infected AIS patients (Figure 1A, *P*=0.0258), while no significant differences were observed for circPDS5B (Figure 1B, *P*=0.9750) and circCDC14A (Figure 1C, *P*=0.7973). To investigate the potential power of circFUNDC1 in predicting SAI among AIS patients, receiver operating characteristic (ROC) curves were constructed to compare the expression levels in SAI patients and not infected AIS patients (Figure 1D), and the area under the curve (AUC) was 0.6612, with sensitivity and specificity at 69.23% and 61.90%, respectively.

**Table 1:** Demographic and baseline clinical characteristics of all stroke patients involved in our study

	Non-SAI	SAI	*P*
**Demographic characteristics**			
**Total, *n***	42	26	
**Age, mean (SEM), y**	70 (61–81)	80 (66–85)	0.032
**Female, *n* (%)**	16 (38.10)	8 (30.77)	0.539
**Vascular risk factors, *n* (%)**			
**Hypertension**	35 (83.33)	16 (61.54)	0.440
**Smoking history**	10 (23.81)	5 (19.23)	0.658
**Atrial Fibrillation**	6 (14.29)	7 (26.92)	0.220
**Diabetes mellitus**	17 (40.48)	7 (26.92)	0.256
**Previous TIA/stroke/MI**	5 (11.90)	6 (23.08)	0.312
**Laboratory parameters, mean (SEM)**			
**Glucose (mmol/l)**	6.24 (5.50–8.77)	6.81 (5.82–10.10)	0.358
**Triglycerides (mmol/l)**	1.19 (0.88–1.68)	0.96 (0.67–1.20)	0.014
**Total cholesterol (mmol/l)**	4.32 (3.76–5.22)	4.75 (3.45-5.09)	0.950
**HDL (mmol/l)**	1.18 (0.94–1.25)	1.14 (0.97–1.42)	0.925
**LDL (mmol/l)**	2.63 (0.13)	2.64 (0.19)	0.982
**Lpa (g/l)**	154 (81–456)	220 (94–384)	0.900
**WBC (10^9^/l)**	7.22 (0.37)	8.75 (0.57)	0.018
**RBC (10^12^/l)**	4.78 (0.13)	4.43 (0.16)	0.074
**Neutrophils ratio (%)**	67.75 (57.33–75.08)	79.8 (66.88–83.14)	0.012
**Hemoglobin (g/l)**	143.41 (3.25)	135.58 (4.52)	0.137
**Platelets (10^9^/l)**	200.41 (9.81)	199.42 (10.31)	0.954
**BUN (mmol/l)**	5.0 (4.2–6.5)	6.3 (4.7–7.1)	0.077
**Creatinine (μmol/l)**	76.46 (3.35)	82.27 (5.82)	0.330
**Total Protein (g/l)**	64.52 (0.78)	64.46 (1.43)	0.995
**Albumin (g/l)**	38.7 (34.8–40.4)	38.0 (34.7–40.5)	0.460
**Medication, *n* (%)**			
**ARBs**	6 (14.29)	1 (3.85)	0.238
**ACEIs**	9 (21.42)	1 (3.85)	0.076
**β-Blocker**	7 (16.67)	1 (3.85)	0.142
**CCBs**	14 (33.33)	10 (38.46)	0.667
**Diuretics**	8 (19.05)	2 (7.69)	0.297
**NIHSS**	8 (6–11)	11 (7–16)	0.012

Abbreviations: ACE I, angiotensin-converting enzyme inhibitor; ARB, angiotensin II receptor blocker; BUN, blood urea nitrogen; CCB, calcium channel blocker; HDL, high-density lipoprotein; LDL, low-density lipoprotein; LPa, apolipoprotein A; MI, myocardial infarction; NIHSS, National Institutes of Health Stroke Scale; RBC, red blood cell; SAI, stroke associated infection; TIA, transient ischemic attack; WBC, white blood cell.

### Building baseline risk models to predict the occurrence of SAI

As prediction of SAI might not be accurately achieved merely by assessing the baseline levels of circFUNDC1. Therefore, we aimed to build risk models based on the baseline characteristics of stroke patients to predict SAI (Table 2). As a first step, we used only age, NIHSS score, WBC and triglycerides level on admission as variables in the logistic regression model A. Subsequently, the level of circFUNDC1 was added to logistic regression model B. We chose these variables since they were independently associated with SAI in the univariate analysis. As a result, older age (odds ratio: 1.035; 95% confidence interval [CI]: 0.991–1.080; *P*=0.120), higher NIHSS (odds ratio: 1.121; 95% CI: 0.978–1.286; *P*=0.101), higher level of WBC (odds ratio: 1.311; 95% CI: 1.023–1.680; *P*=0.032) and lower level of triglycerides (odds ratio: 0.503; 95% CI: 0.179–1.413; *P*=0.192) on admission were correlated with SAI in model A. Similarly, older age (odds ratio: 1.036; 95% confidence interval [CI]: 0.991–1.082; *P*=0.116), higher NIHSS (odds ratio: 1.116; 95% CI: 0.973–1.280; *P*=0.116), higher level of WBC (odds ratio: 1.278; 95% CI: 0.995–1.642; *P*=0.055) and lower level of triglycerides (odds ratio: 0.549; 95% CI: 0.193–1.564; *P*=0.262) were involved in predicting outcomes in model B. Though the baseline increasing level of circFUNDC1 (odds ratio: 1.000; 95% CI: 1.000–1.001; *P*=0.294) only slightly associated with SAI in model B, it helps to increase AUC of model A from 0.7971 to 8038 in model B according to ROC curve in Figure 1E,F. Moreover, model B helps minimize false-positive rate with higher specificity from 62.5% in model A to 67.5% in model B.

**Table 2:** Risk models to predict SAI after stroke

	Risk model A			Risk model B		
	OR	95%CI	*P*-value	OR	95%CI	*P*-value
**Age**	1.035	0.991–1.080	0.120	1.036	0.991–1.082	0.116
**Triglycerides (mmol/l)**	0.503	0.179–1.413	0.192	0.549	0.193–1.564	0.262
**WBC (10^9^/l)**	1.311	1.023–1.680	0.032	1.278	0.995–1.642	0.055
**NIHSS**	1.121	0.978–1.286	0.101	1.116	0.973–1.280	0.116
**circFUNDC1**	/	/	/	1.000	1.000–1.001	0.294

### Kaplan–Meier curves estimates SAI and survival for AIS patients

**Figure 2:**
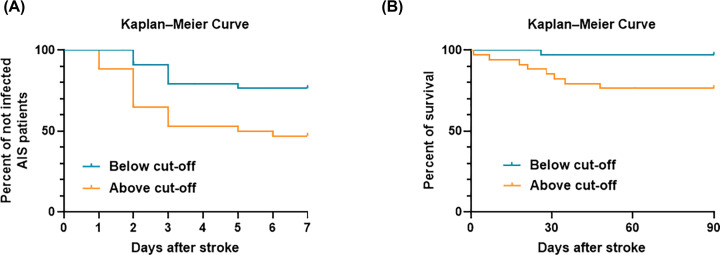
Kaplan–Meier curves estimates SAI and survival for AIS patients (**A**) Kaplan–Meier curves estimates SAI during the first 7 days after stroke, *n*=34/34, *P*=0.0078, Log rank test. (**B**), Kaplan–Meier curves estimates survival till to 90 days after stroke, *n*=34/34, *P*=0.0129, Log rank test.

To further verify the significance of circFUNDC1 in predicting SAI and stroke outcome. Cut off value of circFUNDC1 expression level from ROC curve (Figure 1B) was defined as ‘727 copies/μl plasma’ which had sensitivity and specificity at 69.23% and 61.90%, respectively. Afterwards, plot Kaplan–Meier curves estimated SAI (days) for patients with low (below cut-off) or high level of circFUNDC1 (above cut-off) were constructed as Figure 2A found the curves of the two groups were significantly different (*P*=0.0078, log rank test). Moreover, Kaplan–Meier curves estimated survival of AIS patients with low (below cut-off) or high circFUNDC1 levels (above cut-off) were constructed after follow up for 3 months, and found the curves of the two groups were significantly different (Figure 2B, *P*=0.0129, log rank test) too.

### Possible cellular source and correlation of circFUNDC1 level and blood cell counts in blood

**Figure 3:**
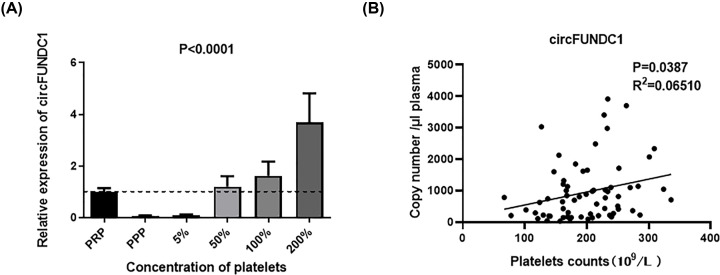
Platelet spike-in experiment as well as the correlation between platelets counts and copy number of circFUNDC1 per microliter plasma (**A**) Levels of circFUNDC1 after spiking back platelets into platelet-poor plasma (PPP) compared with platelet-rich plasma (PRP), *n*=8. Mean ± SEM, Friedman test. (**B**) Correlation between platelet counts and copy number of circFUNDC1 per microliter plasma; *N*=66, Pearson correlation analysis.

Previous studies have demonstrated that platelets are an important source of circulating circRNAs [14]. As platelets were reported to be relatively abundant in circRNAs [15], we aimed to determine whether circFUNDC1 was enriched in platelets. Accordingly, we separated platelets from platelet-rich plasma and spiked them back into platelet-poor plasma (PPP) at increasing concentrations [13]. Compared with the level of circFUNDC1 in PPP, the expression levels of circFUNDC1 significantly increased with increasing concentrations of platelets (Figure 3A). Moreover, copy number of circFUNDC1 per microliter plasma was positively correlated with the number of platelets (Figure 3B), indicating that circFUNDC1 were enriched in platelets.

**Figure 4:**
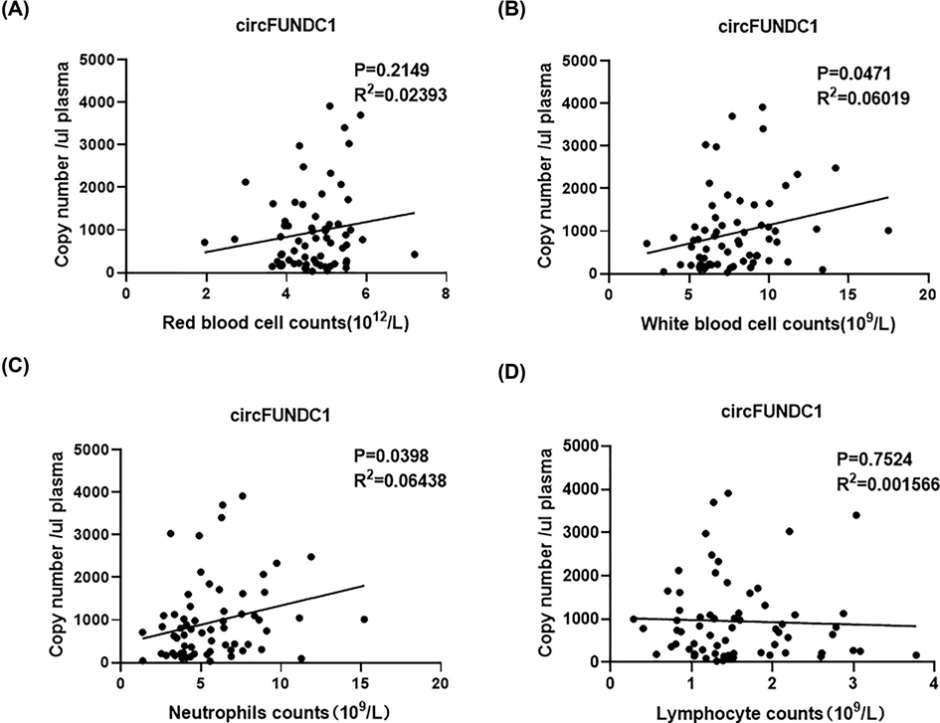
Correlations between circFUNDC1 levels and red blood cell (RBC), white blood cell (WBC), neutrophil and lymphocyte counts (**A**) Correlation between circFUNDC1 levels and RBC counts. (**B**) Correlation between circFUNDC1 levels and WBC counts. (**C**) Correlation between circFUNDC1 levels and neutrophil counts. (**D**) Correlation between circFUNDC1 levels and lymphocyte counts; *N*=66, Pearson correlation analysis.

Additionally, red blood cell (RBC) and WBC were imperative ingredients of peripheral blood cells and could be origins of circFUNDC1, too. However, no significant correlation was found between RBC counts and circFUNDC1 level (Figure 4A, *P*=0.2149, *R*^2^=0.02393). As WBCs were critical for peripheral immune system, correlation analysis was conducted between WBC counts and circFUNDC1 level, finding positive correlation between them (Figure 4B, *P*=0.0471, *R*^2^=0.06019). As two most important ingredients in WBC, neutrophil counts were significant correlated with circFUNDC1 level in plasma (Figure 4C, *P*=0.0398, *R*^2^=0.06438), while lymphocyte counts were not (Figure 4D, *P*=0.7524, *R*^2^=0.001566). Additionally, WBC and neutrophils ratio were elevated in SAI patients compared to non-SAI patients (Table 1), indicating neutrophils might be important origins of elevated circFUNDC1 level in plasma for SAI patients.

## Discussion

The main finding of the present study is that in addition to age, NIHSS score and WBCs, circFUNDC1 level was newly found to be associated with the occurrence of SAI. Particularly, when adding circFUNDC1 into risk models in predicting SAI, the prediction significance increased with increased specificity. Moreover, neutrophils might be important origins of elevated circFUNDC1 levels in plasma among SAI patients though the detailed mechanisms are remains to be uncovered.

As reported in our previous research, circFUNDC1 has been verified to be important biomarker in stroke diagnosis and prognosis [12]. Our present study furtherly illustrated that the incidence of SAI increased with increasing level of circFUNDC1. Previous researches have demonstrated that circRNAs can function in gene regulation by competing with linear splicing [16]; thus, circFUNDC1 could function through their host genes after stroke. The host gene of circFUNDC1-FUNDC1 has been verified to play an imperative role in mitophagy. Ablation of the mitophagy receptor FUNDC1 leads to impaired mitophagy, which might partially account for deformed mitochondria and pronounced oxidative stress [17]. Though the precise contribution of FUNDC1 to basal or stimulated (by stress of stroke) mitophagy activity in peripheral blood cells remains undetermined, we hypothesize that mitophagy might be an important aspect of worsening outcomes in stroke patients, especially in peripheral neutrophils that are closely associated with peripheral immune system. Therefore, circFUNDC1 could be a potential treatment target of SAI according to these mechanisms mentioned above, while further research is needed to verify these assumptions in the future.

As for the origins of elevated circFUNDC1 in plasma, our previous research has shown that no significant increase of circFUNDC1 levels was found in platelets and granulocytes of AIS patients compared with healthy controls [12]. However, the current study demonstrated that circFUNDC1 was enriched in platelets meaning that platelets might be important origins of circFUNDC1 in plasma. Moreover, neutrophils were verified to be important origins of elevated circFUNDC1 level in SAI patients partially owing to neutrophils affect the immune state of stroke patients, contributed to the onset of stroke associated infection.

To sum up, the present study was novel in identifying the significance of circRNA as predictor of SAI. The strengths of our research were listed as the following: (1) Only AIS patients with NIHSS score between 5 to 20 were involved because these patients were at high risk of SAI and the prevention medications might be more valuable for them; (2) Despite only studying the value of circFUNDC1 in SAI prediction, we also analyzed their value together with other significant characteristics in predicting SAI; (3) Possible origins of circFUNDC1 were identified by correlation analysis with peripheral blood cells.

However, our study also has some limitations. First, only 68 patients were involved in our study, meaning that more AIS patients should be included in our study to further verify our findings. Second, the predictive significance of circFUNDC1 level for SAI was relatively weak, while it decreased false-positive rate when compounding with NIHSS, age, WBCs. Finally, though platelets and neutrophils might be important origins of circFUNDC1 in plasma, the precise mechanism need to be furtherly explored.

In conclusion, we were the first to explore the value of circulating circFUNDC1 in predicting SAI among AIS patients at risk. Our investigation showed that circFUNDC1 together with NIHSS, age, WBCs could be used to construct a risk model for SAI prediction.

## Perspectives

- CircFUNDC1 has been reported to be diagnosis and prognosis biomarker of AIS.
- CircFUNDC1 is beneficial in predicting SAI and could be used to construct a risk model together with other risk factors.
- The increased significance of models in predicting SAI could help to guide preventive treatment in stroke patients and circFUNDC1 might provide new insights into therapeutic targets for SAI.

## Abbreviations

ACE I: angiotensin-converting enzyme inhibitor
AIS: acute ischemic stroke
ARB: angiotensin II receptor blocker
AUC: area under the curve
BUN: blood urea nitrogen
CCB: calcium channel blocker
circFUNDC1: circular RNA FUNDC1
HDL: high-density lipoprotein
LDL: low-density lipoprotein
MI: myocardial infarction
NIHSS: National Institutes of Health Stroke Scale
qPCR: quantitative real-time polymerase chain reaction
ROC: receiver operating characteristic
SAI: stroke associated infection
TIA: transient ischemic attack
WBC: white blood cell
